# Treatment with an orally bioavailable prodrug of 17β-estradiol alleviates hot flushes without hormonal effects in the periphery

**DOI:** 10.1038/srep30721

**Published:** 2016-08-01

**Authors:** Istvan Merchenthaler, Malcolm Lane, Gauri Sabnis, Angela Brodie, Vien Nguyen, Laszlo Prokai, Katalin Prokai-Tatrai

**Affiliations:** 1 Department of Epidemiology & Public Health and Anatomy & Neurobiology, University of Maryland, Baltimore, MD 21154, USA; 2 Department of Pharmacology, University of Maryland, Baltimore, MD 21154, USA; 3 Center for Neuroscience Discovery, Institute for Healthy Aging, University of North Texas Health Science Center, Fort Worth, TX 76107, USA

## Abstract

Estrogen deprivation has a profound effect on the female brain. One of the most obvious examples of this condition is hot flushes. Although estrogens relieve these typical climacteric symptoms, many women do not want to take them owing to unwanted side-effects impacting, for example, the uterus, breast and blood. Therefore, there is a need for developing safer estrogen therapies. We show here that treatment with 10β,17β-dihydroxyestra-1,4-dien-3-one (DHED), a novel brain-targeting bioprecursor prodrug of the main human estrogen, 17β-estradiol, alleviates hot flushes in rat models of thermoregulatory dysfunction of the brain. Oral administration of DHED elicits a significant reduction of tail skin temperature (TST) rise representing hot flushes in the morphine-dependent ovariectomized rat model and results in the restoration of estrogen deprivation-induced loss of diurnal rhythm in TST. These beneficial effects occur without detrimental peripheral hormonal exposure; thus, the treatment avoids potentially harmful stimulation of estrogen-sensitive peripheral organs, including the uterus and the anterior pituitary, or the proliferation of MCF-7a breast cancer cell xenografts. Our promising preclinical assessments warrant further considerations of DHED for the development of a brain-selective 17β-estradiol therapy to relieve hot flushes without undesirable peripheral side-effects.

One of the best demonstrations of how estrogen deprivation affects brain function is the peri-menopausal hot flushes due to sudden decline in circulating estrogen levels. Hot flushes are the most common and characteristic symptoms associated with the female climacterics and they negatively affect the quality of life. They represent the most prevalent neurological symptom for which women seek remediation^1^,^2^. The mechanism of hot flushes is not well understood. It has been suggested that a slight elevation in core body temperature is sensed by heat-sensitive neurons in the hypothalamus (*thermostat*), which activates the descending sympathetic nervous system to dissipate heat by vasodilation and sweating (*hot flush*)^3^. Some studies in rats suggest that the sensitivity of these thermoregulatory neurons is affected by estrogens, norepinephrine, serotonin^4^ and neurokinin B^5^. Although hot flushes are characteristic events of primates, the close association between estrogens and thermoregulation makes rodent models useful to evaluate the utility of novel approaches against hot flushes. In addition to the predominant hypothalamic mechanisms, tail skin temperature (TST) regulation may also involve local estrogen receptors^6^. It has been shown that estrogens prevented the ovariectomy- (OVX-) induced TST changes in two established animal models of hot flushes; in the morphine-dependent pharmacological model^7^ and in a “physiological” model involving estrogen-dependent diurnal oscillation of TST^4^,^8^.

Among current therapies, only estrogen (alone or combined with a progestin) can alleviate hot flushes completely, but many women cannot or do not want to take estrogens because of their potential side effects. These side effects occur in the periphery, including in the uterus and in the breast, where estrogen may stimulate cell proliferation and even malignancy. High levels of circulating estrogens may also have cardiovascular liability causing deep venous thrombosis with subsequent pulmonary embolism^9^,^10^. Therefore, many women turned to non-steroid-based therapies including soy products, serotonin and norepinephrine reuptake inhibitors, clonidine, and gabapentine^11^,^12^,^13^,^14^. The efficacy of these alternate therapies, however, is minimal—usually just above the placebo effect and they can also carry side effects. Hence, there is an unmet need for developing efficacious estrogen therapies with improved safety profile. A plausible solution to this challenge is to confine the beneficial effect of estrogens into the brain *via* a unique prodrug design. A prodrug is inert and remains inactive until *in vivo* bioconversion to the active agent^15^.

We have recently reported that a small-molecule compound, 10β,17β-dihydroxyestra-1,2-dien-3-one (DHED), is an inactive bioprecursor prodrug of 17β-estradiol (E_2_) converting to E_2_ only in the brain^15^. This prodrug also possesses favorable physicochemical properties for blood-brain barrier transport compared to those of E_2_. Therefore, utilizing DHED as an E_2_-bioprecursor may be a viable approach for delivering E_2_ selectively into the brain for the potential treatment of hot flushes. In this study, we report that DHED treatment profoundly reverses the OVX-induced TST changes in two rodent models of hot flushes without exposing the periphery to the hormone after the clinically preferred oral (p.o.) administration.

## Results

### DHED treatment significantly increases E_2_ concentration in the hypothalamus without increasing circulating E_2_

**Figure 1:**
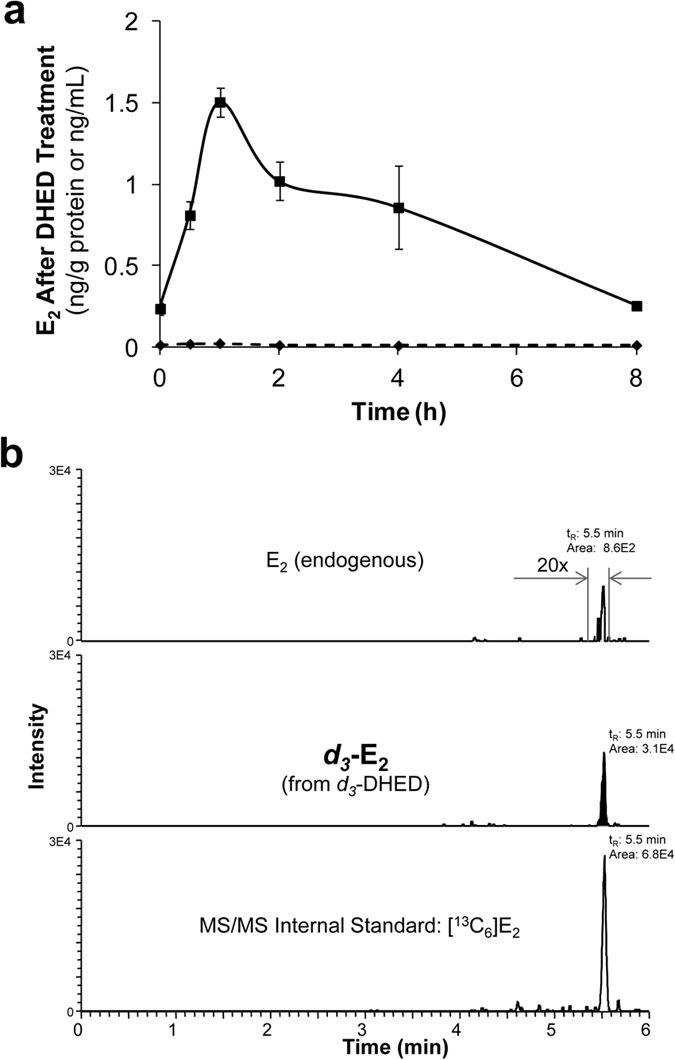
DHED treatment produces E_2_ in the hypothalamus. (**a**) After DHED treatment of OVX Sprague-Dawley rats (200 μg/kg, p.o.), E_2_ concentration increases in the hypothalamus but not in the serum, as measured by LC-MS/MS assay (n = 3–4 rats/time point). Data represent mean ± SEM. (**b**) Administration of a deuterium-labeled (*d*_*3*_-) DHED, produces *d*_*3*_-E_2_ (middle trace) in the hypothalamus without influencing endogenous E_2_ level (top trace). The chart shows LC-MS/MS analyses from tissue harvested 1 h after *d*_*3*_-DHED (200 μg/kg, p.o.), using [^13^C_6_]E_2_ added to the tissue homogenate as an analytical reference.

In the present study, we concentrated on this highly relevant section of the brain where the heat-sensitive neurons are located^3^. As shown in Fig. 1a, liquid chromatography-tandem mass spectrometry (LC-MS/MS) measurements^16^ revealed a significant increase in E_2_ levels in the hypothalamus of OVX rats treated with DHED (p.o., 200 μg/kg). Maximum E_2_ concentration was reached about 1 h after the administration of the prodrug, and the mean residence time of the DHED-derived E_2_ in the hypothalamus was about 2.5 h at this dose according to non-compartmental pharmacokinetic analysis^17^. By administering a deuterium-labeled form of the prodrug (*d*_*3*_-DHED; 200 μg/kg, p.o.), we also established by our LC-MS/MS assay developed to distinguish different isotopic forms of E_2_ that *d*_*3*_-DHED produced *d*_*3*_-E_2_ in the hypothalamus without elevating endogenous E_2_ concentration (Fig. 1b). No detectable level of *d*_*3*_-E_2_ was found in the blood, supporting the brain-selective formation of estrogen from the prodrug.

### Effect of DHED treatment in the pharmacological model of hot flushes

**Figure 2:**
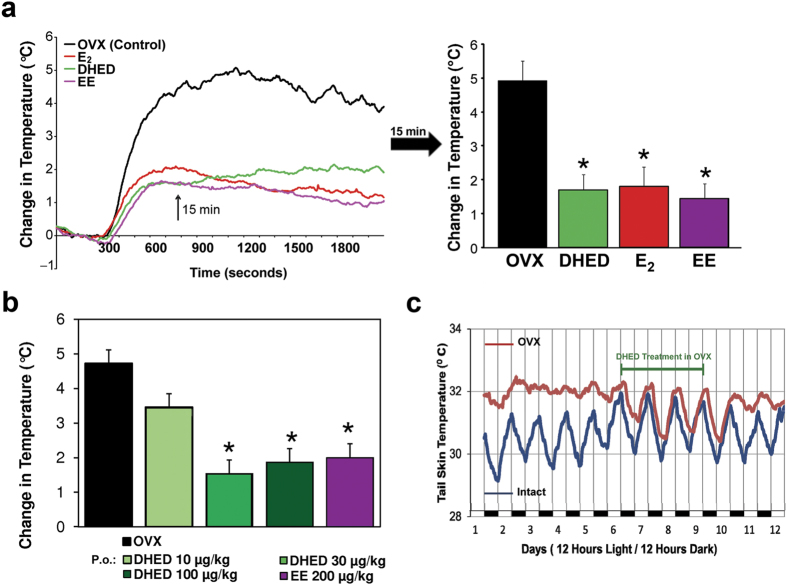
Administrations of estrogens (E_2_ and/or EE) and DHED reduce naloxone-induced TST rise in morphine-dependent OVX rats. Orally administered DHED also restores rhythmic oscillation of TST in OVX rats. (**a**) The effect of s.c. treatments on TST. Left side: TST starts to elevate 5 min after naloxone treatment and reaches peak values between 10–15 min, after which TST drops and eventually returns to baseline by 60 min (not shown). While naloxone withdrawal induces a 5 °C increase in TST of OVX-vehicle-treated rats (black trace), E_2_ (red trace), EE (magenta trace) and DHED (green trace) blunt this rise to less than 2 °C. Chart on the right: TST values measured at 15 min after naloxone injection (arrow). All three treatments blunted TST rise compared to OVX-vehicle-treated animals. Data represent mean ± SEM. *P < 0.01 compared with vehicle-treated OVX group. (**b**) The effects of orally administered DHED (10, 30 or 100 μg/kg), EE (200 μg/kg), or vehicle on the mean changes in TST. Both DHED (shades of green, increasing dose reflected by darker color) and EE (magenta) blunt TST rise compared to OVX-vehicle-treated animals (black). Data represent mean ± SEM. *P < 0.05 compared with vehicle-treated OVX group. (**c**) The effect of DHED administered orally (50 μg/kg b.i.d.) on diurnal TST changes. The TST of intact animals (blue line) show rhythmic changes during the active and passive phases of the day. The TST of OVX rats (red line) does not show diurnal changes. However, when OVX rats are treated with DHED from day 5 through 9, the diurnal changes return to those seen in intact rats. When DHED treatment is terminated on day 10, the undulating TST changes disappear.

In the first experiment, DHED (20 μg/kg), ethinyl estradiol (EE, 200 μg/kg) or E_2_ (200 μg/kg) were administered subcutaneously (s.c.) for ten days. DHED dose in this experiment was chosen based on previous results demonstrating that the s.c. dose of the prodrug required to elicit the same neuroprotective effect was about 10-times less than that of the parent estrogen (E_2_)^15^. As Fig. 2a shows, TST began to rise 5 min after naloxone injection, reached its highest level of increase (approximately + 5 °C) 15 min after morphine withdrawal; then, TST declined and returned to baseline within 45–60 min. Since the rise in TST was greatest 15 min after naloxone injection (arrow in Fig. 2a), data collected at this time point were analyzed for statistical differences and potency estimation. All three test compounds similarly attenuated the TST rise evoked by morphine withdrawal compared to the vehicle-treated OVX animals (Fig. 2a). In the second experiments, dose-response studies were done by using 10, 30, and 100 μg/kg p.o. DHED treatments, twice daily, for ten days. This resulted in a dose-dependent suppression of the rise of TST evoked by morphine withdrawal. The highest elevation in TST was seen in the vehicle-treated rats while the lowest elevations were observed in the DHED- (30 and 100 μg/kg) and EE-treated animals (Fig. 2b). The later clinically used agent served as a positive control in these studies.

### Effect of DHED treatment on diurnal TST changes (physiological model)

Tail skin temperature of intact, sham-operated rats changes during the active and passive phases of the day^4^,^8^,^18^,^19^,^20^. As Fig. 2c shows, TST fluctuated around 31.7 °C during the passive (lights on) phase, whereas it varied around 29.7 °C in the active (lights off) phase. In OVX animals, TST during the passive (lights on) and active (lights off) phases were similar; i.e., the rhythmic fluctuation present in intact animals disappeared. Moreover, TSTs of OVX rats were not only flat, but somewhat elevated (32.2 °C compared to 30.5 °C in intact female rats). Similarly to our previous observations with EE^4^ and others with E_2_^8^, orally administered DHED at a dose of 2-times 50 μg/kg/day completely restored the rhythmic changes in TST lost in OVX rats. When the treatment with the prodrug ceased, the fluctuation disappeared.

### Effect of DHED treatment on progesterone receptor expression in the brain

**Figure 3:**
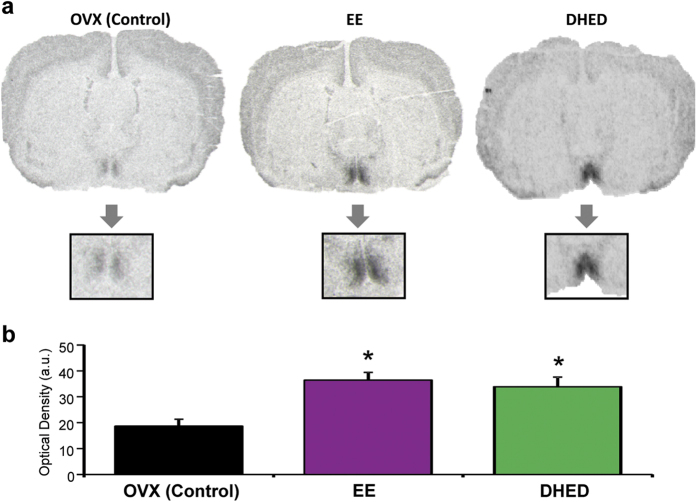
DHED treatment induces PR expression, an estrogen marker in the hypothalamus. (**a**) DHED administration (p.o., 30 μg/kg once a day for 4 days) induces PR expression, evaluated with *in situ* hybridization histochemistry in the POA of the animals tested in the physiological model of hot flushes (Fig. 2b). Vehicle and EE (p.o., 200 μg/kg once a day for 4 days) were included in the experimental design as negative and positive (estrogen) controls, respectively (n = 4–6 per treatment group). (**b**) Optical density over the POA in DHED, EE or vehicle-treated rats. Both EE and DHED significantly increased optical density, representing PR expression in the POA compared to vehicle-treated animals. Data represent mean ± SEM. *P < 0.05 compared with vehicle-treated OVX group.

DHED-derived E_2_ stimulated the expression of progesterone receptor (PR) in the preoptic area (POA) of the hypothalamus, shown by *in situ* hybridization histochemistry^21^,^22^. The effect of DHED on PR expression has also been evaluated on animals not involved in the hot flush studies to avoid morphine as a potential confounder. Here, we show that PR expression in brains taken from morphine-treated rats manifested the same response to DHED or EE as in OVX rats not treated with morphine (Fig. 3a). These animals were treated with the test compounds (DHED 30 and 100 μg/kg, EE 200 μg/kg, and vehicle) *via* oral gavage twice per day. Since our previous observations showed that DHED at 30 μg/kg was as efficacious as at 100 μg/kg in the hot flush model (data not shown), here we only evaluated the effect of 30 μg/kg DHED on PR expression. We found, that induction of this highly estrogen-responsive gene, as indicated by optical density on film exposures, was three fold higher in the DHED- and EE-treated groups than in the vehicle-treated animals (Fig. 3b).

### Effect of DHED treatment on estrogen-sensitive peripheral tissues

**Figure 4:**
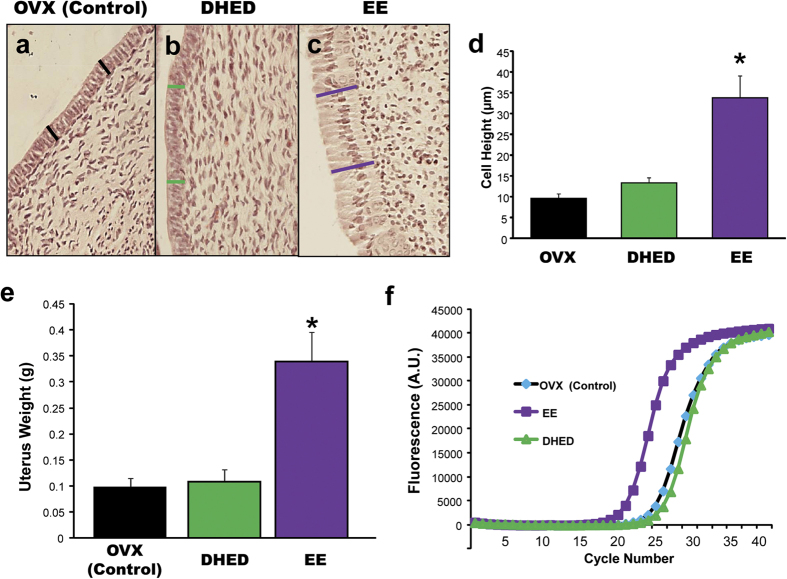
DHED treatment is not accompanied by typical estrogenic effects in the periphery . (**a**–**d**) DHED, contrary to EE, does not stimulate the uterus; i.e., it does not increase epithelial cell height. Data represent mean ± SEM. *P < 0.05 compared with vehicle-treated OVX group. (**e**) DHED, contrary to EE, does not stimulate uterine wet weight. (**f**) DHED, contrary to EE does not stimulate the expression of galanin in the anterior pituitary as measured with quantitative reverse transcriptase polymerase chain reaction (qRT-PCR). Data represent mean ± SEM. *P < 0.001 compared with vehicle-treated OVX group.

**Figure 5:**
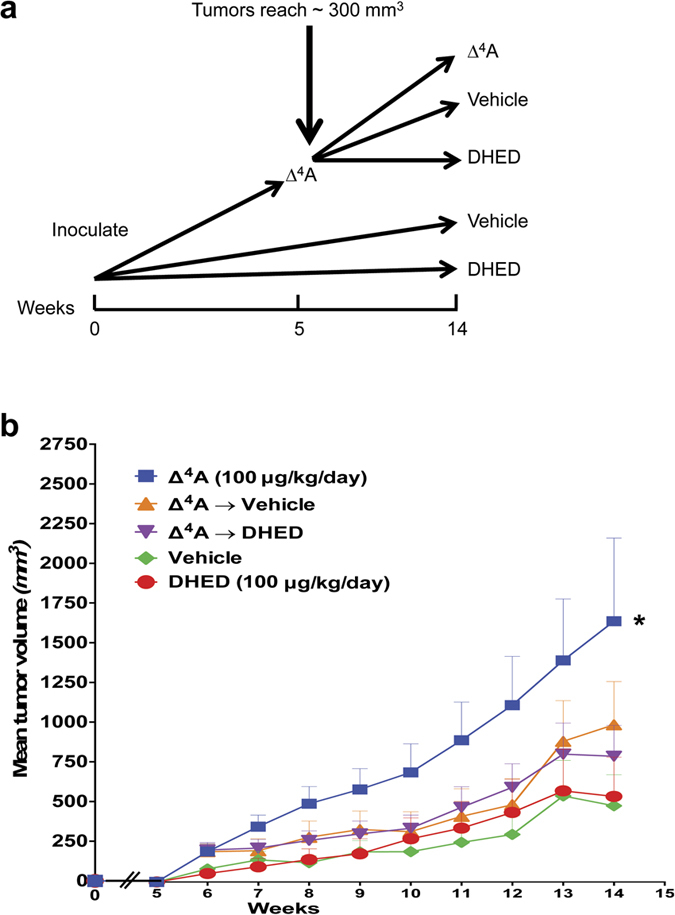
DHED treatment does not induce proliferation of xenografts containing MCF-7Ac-1a breast cancer cells. (**a**) MCF-7Ac-1a breast cancer cells were injected into nude mice and left proliferate for 5 weeks. Then, 30 mice were treated with Δ^4^-androstenedione (Δ^4^A), 10 with vehicle and 10 with DHED. At the beginning of week 6, the treatment of 10 mice from 30 continued with Δ^4^A, the treatment in 10 mice was switched to vehicle and in 10 mice to DHED and the treatment continued for an additional 10 weeks. The tumor sizes were measured daily and at the time of euthanasia. (**b**) Tumors treated with Δ^4^A grew fast and the main tumor volume reached 1750 mm^3^ by the 14^th^ weeks. Tumors originally treated with Δ^4^A, then switched to vehicle or DHED proliferated slower than those treated with Δ^4^A only and the rate of proliferation in these groups was similar to cells treated with vehicle or DHED only. There were no significant differences among vehicle-, DHED-, Δ^4^A-vehicle-, and Δ^4^A-DHED-treated groups However, the proliferation was significantly higher in the Δ^4^A alone group compared to all the other groups. Data represent mean ± SEM; *P < 0.05 compared with vehicle-treated OVX group.

We focused our studies on the uterus, anterior pituitary, and cancerous breast cells as representative peripheral organs highly sensitive to the presence of exogenously administered estrogens. Uteri were taken from the animals used in the above studies. DHED, even at the highest dose (100 μg/kg per day) did not produce statistically significant proliferation of the epithelial cells of the endometrium (Fig. 4a–d) and also did not increase uterine wet weight (Fig. 4e). Expectedly, EE significantly increased wet weight and endometrial cell height of the uterus (Fig. 4a–e). Contrary to EE treatment, DHED administration also did not induce the expression of the galanin gene, which is highly regulated by estrogens^23^,^24^ in the anterior pituitary (Fig. 4f). Similarly, the use of the prodrug did not stimulate tumor cell growth in aromatase-transfected MCF-7Ca breast cancer cells^25^ compared to vehicle (Fig. 5). While the tumor expectedly grew intensely until the end of the experiments in the animals treated with Δ^4^-androstenedione (Δ^4^A, the substrate of the aromatase making estrogen), the proliferation of cancer cells originally treated with Δ^4^A and, then, with DHED slowed down to a level close to what was seen in the vehicle or DHED-alone treatment groups.

## Discussion

We have recently reported that DHED, independently of the routes of administration, converts to E_2_ in the brain of OVX rats but not in the periphery^15^. Therefore, appreciable increase in circulating E_2_ could not be detected even by our sensitive LC-MS/MS assay^16^. On the other hand, a significant boost in E_2_ level in the hypothalamus, the thermoregulatory center of the body^26^ and thus, highly relevant in the present context, was established due to the brain-selective formation of E_2_ from the prodrug after p.o. administration (Fig. 1a). Following the bioconversion of a stable-isotope-labeled form of the prodrug (*d*_*3*_-DHED), *d*_*3*_-E_2_ was shown to form in the brain without triggering endogenous E_2_ formation through the aromatase-catalyzed pathway^15^ or without the appearance of *d*_*3*_-E_2_ in the circulation.

As a consequence of E_2_ formation from the prodrug in the brain, administration of DHED reduced TST rise in the morphine-dependent hot flush model, restored the OVX-induced loss of rhythmic, diurnal TST changes, and induced the expression of PR in the POA of the hypothalamus. PR expression is highly stimulated by E_2_^21^,^22^ and, therefore, we used this gene as a critical marker for the increase of estrogen in the brain. As such, PR expression was an independent confirmation of what we have quantitatively established by our validated and highly reproducible LC-MS/MS assay (Fig. 1) in terms of E_2_ formation from DHED in the brain. On the other hand, DHED did not produce estrogenic effects in the periphery, as no statistically significant increase in circulating E_2_ level compared to that of OVX control could be shown. Consequently, DHED administration also did not stimulate the expression of galanin in the anterior pituitary^23^,^24^, whereas E_2_ up-regulated its expression by several folds (Fig. 4f). Concomitantly, unlike EE, the use of DHED did not stimulate the proliferation of epithelial cells in the endometrium of the uterus (Fig. 4a–d) or increase uterine wet weight (Fig. 4e) even at doses that were three times higher than the doses needed to block TST rise or induce profound PR expression in the brain. This finding is important, as women with intact uterus need to take progestin when on estrogen therapy to counterbalance the proliferative effect of estrogen on the uterus. DHED treatment also did not stimulate the proliferation of MCF-7Ac-1 breast cancer xenographts transplanted in nude mice (Fig. 5). Another important finding of our study reported here was that, considering doses necessary to achieve alleviation of hot flushes in an animal model, DHED treatment surpassed the effect of direct E_2_ or EE administration. This was due to DHED’s favorable physiochemical properties (e.g., more water-soluble, less lipophilic, and has decreased binding to plasma proteins) compared to E_2_ and EE^15^, which apparently facilitated brain uptake from the circulation.

The bioreversible removal of the phenolic A-ring from the E_2_ structure upon creating this bioprecursor prodrug also resulted in excellent oral bioavailability of DHED^15^. These findings potentially make DHED a candidate for drug development due to its potency compared to the direct administration of E_2_ even using parenteral (such as s.c.) route. The use of DHED instead of E_2_ having negligible oral bioavailability or orally bioavailable synthetic estrogens such as EE may also offer increased safety owing to brain-selectivity. In the hot flush models, treatment using 30 μg/kg DHED blunted TST rise as efficiently as 200 μg/kg EE when administered orally or as efficiently as 200 μg/kg E_2_ when administered s.c. We have previously shown^7^ that EE administered orally does not alleviate TST rise consistently at doses below 200 μg/kg, and E_2_ has no effect below 100 μg/kg dose when administered s.c. Interestingly, PR induction requires much lower estrogen doses than those necessary for reducing naloxone-induced TST rises.

Estrogens may regulate the activity of thermo-sensitive neurons in the hypothalamus *via* the classical nuclear ERs (ERα or ERβ) or membrane-associated receptors. The potential involvement of both nuclear ERs in thermoregulation and hot flushes has been shown in ER knockout mice^27^ indicating that that the expression of either ERα or ERβ alone can control TST by estrogen. Currently, no data are available on the effect of the membrane-associated ER on thermoregulation and hot flushes. Although the ER phenotype of thermo-sensitive neurons in the POA, the primary thermoregulatory center, has not been established, the colocalization of the two receptors in neurons of the central thermostat^28^ suggests for the involvement of both receptors. Pharmacological studies utilizing the ERα- and ERβ-selective ligands also indicated a role for both receptors in thermoregulation^27^,^29^, although some groups ruled out the role of ERβ in this function^30^,^31^. When evaluating the effect of ER-selective ligands on thermoregulation using rat models, one has to keep in mind that, in addition to acting in the hypothalamus, E_2_ may also act on tail arteries through the local ERβ, which are directly involved in TST regulation^6^. However, our data with the centrally-acting DHED clearly show that hot flushes are centrally initiated events since the DHED-to-E_2_ bioconversion did not produce E_2_ in the periphery and, yet, DHED-derived E_2_ alleviated hot flushes and restored diurnal, rhythmic oscillations of TST, even upon oral administration.

Collectively, based on the present studies and data published recently^15^, we propose DHED as a preclinical drug candidate for the treatment of menopausal hot flushes. DHED may have the potential to be the first efficacious brain-selective estrogen therapy with inherent safety (i.e., no significant liabilities in the periphery) to improve the quality of life of millions of women in the perimenopause and menopause.

## Methods

### Chemicals

DHED and *d*_*3*_-DHED were prepared as reported previously^32^,^33^. Ethinyl estradiol (EE), 17β-estradiol (E_2_) and naloxone were purchased from Sigma (St. Louis, MO, USA). Morphine pellets (75 mg morphine sulfate) were obtained from Murty Pharmaceuticals (Lexington, KY). ^13^C-labeled 17β-estradiol ([^13^C_6_]E_2_) with an isotopic purity of 99% were supplied by Cambridge Isotope Laboratories (Andover, MA, USA).

All animal experimental protocols using Sprague-Dawley rats were approved by the institutional ethics committees of the University of Maryland Baltimore, Baltimore, MD and the University of North Texas Health Science Center, Fort Worth, TX and all experiments were carried out strictly following the guidelines set for the usage of animals by these committees.

### Measurement of E_2_ in the hypothalamus after oral administration of DHED to OVX rats

OVX Sprague-Dawley rats were treated with DHED (200 μg/kg, p.o.) and were euthanized 0.5, 1, 2, 4 and 8 h after treatment. Three to four animals per time point were used. The hypothalami were dissected from the harvested brains, followed by tissue homogenization, adding ^13^C-labeled internal standards (([^13^C_6_]E_2_) and liquid-liquid extraction. The dansyl-derivatized analytes were quantified by isotope-dilution LC–MS/MS as reported previously^16^. The experiment involving oral administration of *d*_*3*_-DHED (200 μg/kg, with euthanasia and tissue harvesting 1 h after treatment) was performed according to a procedure published recently^15^.

### Direct TST measurements in the pharmacological model of hot flush

Experiments were carried our using our previously reported model of hot flushes^7^. In the first experiment, four groups of OVX animals were treated s.c. with DHED (20 μg/kg), vehicle, EE (200 μg/kg), or E_2_ (200 μg/kg). In the second experiment, five groups of OVX rats were treated with DHED (10, 30 or 100 μg/kg), vehicle (negative control), or EE (200 μg/kg; positive control) by oral gavage, twice daily. In both experiments, treatment started 5 days after OVX. In both cases, on the 6^th^ day of treatment, a morphine pellet was implanted s.c. On the 8^th^ day of treatment, two additional morphine pellets were implanted s.c. On the 11th day, the animals were injected with ketamine (80 mg/kg, i.m.) and a thermocouple, connected to a data acquisition system (PhysioTel, Data Sciences International, St. Paul, MN) was taped on the tail approximately one inch from the root of the tail. This system allows the continuous measurement of tail skin temperature. Baseline temperatures were measured for 5–10 min, then naloxone (1.0 mg/kg) was given s.c. (0.2 mL) to block the effect of morphine and TST was measured for 30–40 minutes thereafter. We have previously established that neither ketamine administration nor the duration of treatment have significant effect on the temperature rise caused by morphine withdrawal or on the suppression of this response by exogenous estrogen^7^. At the end of the experiments, rats were euthanized and the brains, uteri and pituitaries were collected and processed as described above.

### Telemetric temperature measurements in the physiological model of hot flush

Under deep anesthesia with isoflurane/oxygen inhalation, a transmitter (PhysioTel TA10TA-F40, Data Sciences International) was implanted s.c. on the back of intact (n = 3) or OVX (n = 8) animals and its temperature sensor was tunneled under the tail skin at a distance of 4–5 cm from the base of the tail^4^,^8^. Following recovery from surgery (6–8 days), the rats, housed individually, were placed on a receiver box and telemetric temperature measurements began for 6 days. Starting on day 6, the OVX animals were treated for 4 additional days with DHED (50 μg/kg) dissolved in physiological saline and administered orally via gavage twice per day between 9:00 and 9:30 am and between 5:00 and 5:30 pm, respectively. The intact animals in the control group were treated with the vehicle only. Temperature values were averaged over 10 seconds for each record; records taken every 3 minutes. A day/night-rhythm was simulated by a “daytime period” of 7 am–7 pm in which room light was switched on, and by a “nighttime period” of the remaining 12 hours where the light was switched off.

### Gene expression studies with *in situ* hybridization histochemistry (ISHH) and RT-PCR

Immediately after finishing TST evaluations in the pharmacological model, the animals were euthanized and their brains were removed and cut with a cryostat to 20-μm thick sections. The sections were placed on gelatin-coated slides, and the expression of PR in the POA of the hypothalamus was evaluated with ISHH utilizing radioactive isotope labeled riboprobe (cRNA) complementary to rat PR sequence according to previously published protocol^21^,^22^. At the end of the hybridization, slides were placed over X-ray films and the optical density of the signal over the POA was evaluated and quantified with C-Imaging (Pittsburg, PA) system. The qRT-PCR protocol followed the procedures reported by Tonelli *et al*.^34^. Briefly, following mRNA extraction with Trizol reagent (Invitrogen, USA), total RNA was treated with DNase (Invitrogen) according to manufacturer’s instructions. Five hundred ng of total RNA per sample were reverse transcribed into cDNA in a 20 μL reaction using an iScript cDNA Synthesis Kit (Bio-Rad, Hercules, CA, USA) according to manufacturer’s instructions. Real-time PCR was performed using the iQ SYBR Green Supermix (Bio-Rad) in a 25 μL reaction using primer pairs for galanin (F 5′ –TCTCACCGCTGCTCAAGATG; R 5′ – GCCATGCTTGTCGCTAAATG) designed with the Accelrys Gene 2.0 v software program. The real-time PCR reaction was run on a MyiQ instrument (Bio-Rad). Efficiency and consistency of the cDNA synthesis was determined by amplification of the 18 S gene as a control. Fold increase was determined using the 2-ΔΔCt method^35^,^36^. According to the Standard Curve Method, the slope of line (−3.32) for a given qPCR along with an efficacy of 1 means that for every 3.32 cycles a 10-fold amplification in the expression of the gene occurs.

### Histology

Uteri were embedded in paraffin and thin (5-μm) sections were stained with hematoxylin-eosin and the height of the epithelial cells was measured at 100X magnification using an Olympus (Exton, PA, USA) microscope and IPLab software.

### Effect in the breast (MCF-7a-c1 cell xenografts)

OVX BALB/c athymic nude mice 4–6 weeks of age were obtained from the National Cancer Institute (Frederick, MD). Subconfluent MCF-7Ac-1 (MCF-7 cells transfected with aromatase) cells were resuspended in Matrigel (10 mg/mL) at 2.5 × 10^7^ cells/mL. Each animal received s.c. inoculations in two sites per flank with 100 μL of cell suspension. Mice were injected s.c. daily with vehicle (n = 10), Δ^4^A-androstenedione (Δ^4^A; 100 μg/day, n = 30), or DHED (100 μg/day, n = 10) in 0.3% (v/v) hydroxypropyl cellulose. Tumor volumes were measured weekly for 14 weeks as reported before^37^. Fifty mice were assigned to groups for treatment so that there were no statistically significant differences in tumor volume among the groups at the beginning of treatment. Mice were treated as described above for 5 weeks. Then, animals in Δ^4^A groups were split into three subgroups: (i) Δ^4^A, (ii) vehicle, or (iii) DHED with ten mice in each group and treatment continued for an additional 10 weeks (Fig. 5a).

### Statistical analyses for the morphine-dependent hot flush model

One- or two-way ANOVA were used followed by appropriate *post hoc* tests to compare differences among groups. P < 0.05 was considered as statistically significant. Data from specific experiments were evaluated as follows: In the morphine-dependent hot flush model, two-factor repeated measures ANOVA model was used to analyze the elevation of TST in morphine-dependent rats. The factors were “treatment” and “time” (repeated). The TST values taken 15 min after naloxone treatment were analyzed for statistical significance. The data were transformed by square root to stabilize the variability. After the transformation, the Huber M-estimation weighting was used to down-weight the outlying transformed observations. The JMP software (SAS Institute, Inc., Cary, NC) was used for the repeated measure analysis of the transformed and weighted observations. Multiple comparisons (LSD P-values) were made among the treatment groups at each time point.

### Statistical analysis for the physiological hot flush model

For the statistical analyses of the diurnal TST changes, we selected 48-h data, starting from 7 a.m. of Day 1 to 7 a.m. of Day 3 in each of the pre-treatment period and after DHED treatment period. For each rat in each period, the mean temperatures at the night and the day were calculated using data from 7 p.m. to just before 7 a.m., and data from 7 a.m. to just before 7 p.m., respectively. A linear mixed model for repeated measures data were performed followed by Tukey’s post hoc test with α  = 0.05. In this model there were two fixed factors (pre-/after-treatment period, and day-night cycle) and the interaction term between the two fixed factors. In addition, we used a compound symmetry covariance structure for the means of temperatures from the same animal, which meant the variances for all mean temperatures were equal and the co-variances for any two mean temperatures in different periods or times from the same animal were equal across different animals. This was equivalent to specifying an animal level random effect.

### Statistical analysis for MCF-7Ac-1 cell growth

Differences in the mean of samples were analyzed using one-way ANOVA with Tukey’s *post hoc* multiple comparisons to establish effect of DHED treatments on MCF-7Ac-1 breast cancer cells. P < 0.05 were considered statistically significant.

## Additional Information

**How to cite this article**: Merchenthaler, I. *et al*. Treatment with an orally bioavailable prodrug of 17β-estradiol alleviates hot flushes without hormonal effects in the periphery. *Sci. Rep*. **6**, 30721; doi: 10.1038/srep30721 (2016).
